# Severe Vertigo After Cochlear Implantation: Acute Pneumolabyrinth

**DOI:** 10.4274/balkanmedj.2017.1088

**Published:** 2018-09-21

**Authors:** Erkan Karataş, Yüksel Toplu, Emrah Gündüz, İsmail Demir

**Affiliations:** 1 Department of Otorhinolaryngology Head and Neck Surgery, İnönü University School of Medicine, Malatya, Turkey

To the Editor,

Pneumolabyrinth is defined as the presence of air within the inner ear including cochlea, vestibule or semicircular canals. This condition is a rare complication of temporal bone fractures, barotrauma and surgeries, such as stapes surgery and cochlear implantation in patients with malformed cochlea (1). We do not expect the occurrence of pneumolabyrinth as an early postoperative complication after cochlear implantation in patients with normal cochlea.

A 34-year-old woman had progressive profound sensorineural hearing loss in both ears. The patient had no vestibular symptoms and had normal otologic examination. Cochlear implantation was performed on the left ear. The computed tomography scan indicated normal middle and inner ear structures with well pneumatized mastoid cells in both ears preoperatively (Figure 1a). Although endoscopic examinations, we did not explore the round window through the posterior tympanotomy in the surgery. We performed promontory cochleostomy anterior superiorly to the possible round window area. The scala tympani was in depth; as such, we drilled deeply to explore it. We also recognized the scala vestibule and found the scala tympani in deep plan. No fluid leakage was observed. We inserted Medel FlexSoft^TM^ electrode (ME-DEL Medical Electronics, Innsbruck, Austria). The patient had severe vertigo after surgery in the early period. We suspected that the electrode was located in the scala vestibuli. Therefore, a computed tomography scan was conducted to check the electrode place postoperatively. The cochlear implant electrode was found in the cochlea. Air shadow was detected in the inner ear, including cochlea, vestibule and semicircular canals (Figure 1b). The patient had horizontal nystagmus to the pathologic ear. Other vestibular tests were not required. The hospitalization time was prolonged for 10 days. We performed conservative management including head elevation, mobilization and vestibular suppression (dimenhydrinate 50 mg intrevenous 4×1/day for 3 days, beta-histidine tablets 24 mg 2×2/day for 1 month). A control computed tomography scan was performed 6 days after the operation (Figure 1c). The amount of air in the cochlea and vestibule in the control scan was lower than that in the second computed tomography scan. The patient manifested reduced symptoms after 10 days and was advised to go home. Vestibular rehabilitation was unnecessary. At present, the patient hears well with the cochlear implant device and is satisfied. Ethics Committee approval and  inform consent were obtained.

The presence of the air in the cochlea and vestibule is typically observed in patients with large vestibular aqueduct syndrome and vestibular schwannoma after cochlear implantation (2,3,4). Pneumolabyrinth after cochlear implantation has been reported in malformed cochlea (3). The preset case differs from those in previous reports. We did not observe the round window through the posterior tympanotomy. We performed promontory cochleostomy anterior superiorly to the possible round window area. The scala tympani was in depth; as such, we drilled deeply to explore it. We also recognized the scala vestibule and found the scala tympani in deep plan. We decided that we damaged the basilar membrane, resulting in the entry of air bubbles to the scala vestibuli.

In conclusion, acute pneumolabyrinth should be considered when patients suffer from severe vertigo after cochlear implantation in the early postoperative period. Such condition could be due to non-malformed cochlea, and promontory cochloestomy is necessary. We should not drill the promontory deeply for cochleostomy and we should not cause possible basilar membrane damage to avoid pneumolabyrinth.

## Figures and Tables

**Figure 1:**
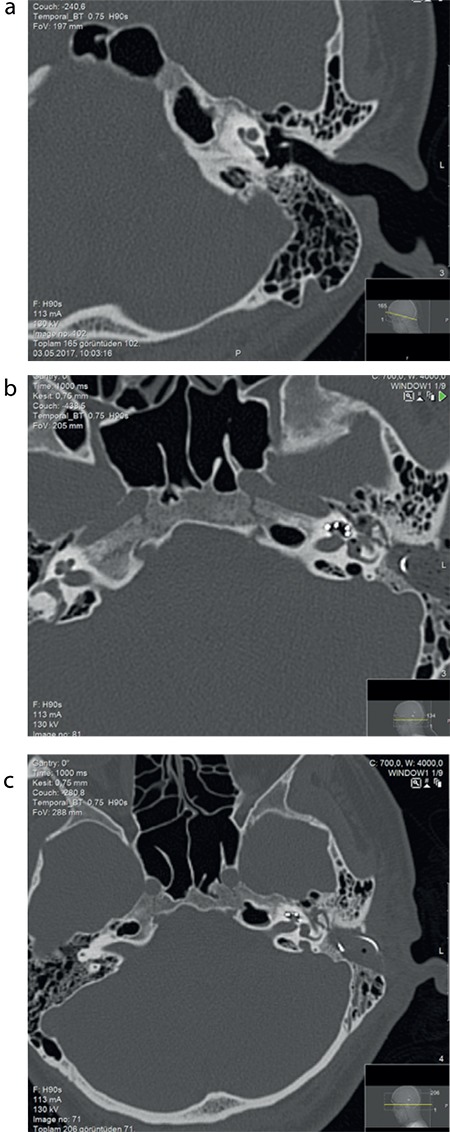
a-c. Computed tomography scan showing normal middle and inner ear structures with well pneumatized mastoid cells in the left ear (a), second computed tomography scan demonstrating the cochlear implant electrode in the cochlea in the early postoperative period. An air shadow was found in the inner ear, including cochlea, vestibule, and semicircular canals. This condition is defined as acute pneumolabyrinth (b), third control computed tomogrphy scan performed 6 days after the operation. The amount of air in the cochlea and vestibule in the control scan was lower than that in the second computed tomography scan (c).
